# The Happiest Kids on Earth. Gender Equality and Adolescent Life Satisfaction in Europe and North America

**DOI:** 10.1007/s10964-017-0756-7

**Published:** 2017-10-11

**Authors:** M. E. de Looze, T. Huijts, G. W. J. M. Stevens, T. Torsheim, W. A. M. Vollebergh

**Affiliations:** 1 Department of Interdisciplinary Social Science, Faculty of Social and Behavioural Sciences, Utrecht University, P.O. Box 80.140, 3508 TC Utrecht, The Netherlands; 2 Department of Sociology, University of York, Wentworth College, W/247, Heslington, YO10 5DD UK; 3 Department of Psychosocial Science, Faculty of Psychology, University of Bergen, Postboks 7807, 5020 Bergen, Norway

**Keywords:** Gender equality, Adolescent life satisfaction, Europe, North America, Social support, Multilevel analysis.

## Abstract

Cross-national differences in adolescent life satisfaction in Europe and North America are consistent, but remain poorly understood. While previous studies have predominantly focused on the explanatory role of economic factors, such as national wealth and income equality, they revealed weak associations, at most. This study examines whether societal gender equality can explain the observed cross-national variability in adolescent life satisfaction. Based on the assumption that gender equality fosters a supportive social context, for example within families through a more equal involvement of fathers and mothers in child care tasks, adolescent life satisfaction was expected to be higher in more gender-equal countries. To test this hypothesis, national-level data of gender equality (i.e., women’s share in political participation, decision making power, economic participation and command over resources) were linked to data from 175,470 adolescents aged 11–16 years old (*M*_*age*_ = 13.6, *SD* = 1.64, 52% girls) from 34 European and North American countries involved in the 2009/10 Health Behaviour in School-aged Children (HBSC) study. Results of linear multilevel regression analyses indicate that adolescents in countries with relatively high levels of gender equality report higher life satisfaction than their peers in countries with lower levels of gender equality. The association between gender equality and adolescent life satisfaction remained significant after controlling for national wealth and income equality. It was equally strong for boys and girls. Moreover, the association between gender equality and life satisfaction was explained by social support in the family, peer and school context. This analysis suggests that gender equality fosters social support among members of a society, which in turn contributes to adolescent life satisfaction. Thus, promoting gender equality is likely to benefit all members of a society; not just by giving equal rights to women and girls, but also by fostering a supportive social climate for all.

## Introduction

Consistent variation in adolescent subjective well-being exists across European and North American countries. In the past decades, the Netherlands and Scandinavian countries have been topping the list, while Eastern European countries tend to score lowest (Bradshaw and Richardson 2009; Cavallo et al. 2015; Inchley et al. 2016). To illustrate, data from the international Health Behavior in School-aged Children (HBSC) study in 2002 (Currie et al. 2004), 2006 (Currie et al. 2008a), 2010 (Currie et al. 2012) and 2014 (Inchley et al. 2016) show that more than 90% of 15-year olds in the Netherlands report high life satisfaction, vs. less than 75% of 15-year old adolescents in Poland. While cross-national differences in sociodemographic factors (e.g., family structure; Bjarnason et al. 2012) are likely to explain some of the cross-national variation, research has increasingly recognized the role of societal factors in explaining the observed cross-national differences in adolescent well-being. Most studies have focused on the explanatory role of economic factors, such as national wealth and income equality, but they revealed weak associations, at most (i.e., adolescents in countries with higher national wealth and more income equality have a slightly higher life satisfaction; Bjarnason et al. 2012; Holstein et al. 2009; Levin et al. 2011a). Much less research has been done on the role of social and cultural factors. Research on adults, however, has clearly revealed the importance of gender equality for adult life satisfaction (Holter 2014; Inglehart et al. 2002; Schyns 1998). It seems likely that gender equality impacts the life satisfaction of the children of these adults as well. Therefore, this study examines the importance of gender equality for adolescent life satisfaction. Based on the assumption that gender equality in society fosters more socially supportive relationships, for example in the family context through more equal involvement of fathers and mothers in child rearing, we expect that adolescent life satisfaction is higher in more gender-equal countries.

### Gender Equality and Life Satisfaction among Adults

In research among adults, gender equality (here defined as the extent to which women and men have an equal share of paid work, money, decision-making power and time in society; Plantenga et al. 2009) has been positively related to life satisfaction (Holter 2014; Inglehart et al. 2002; Schyns 1998). In a large, cross-national study among European countries, Holter (2014) revealed that “the chance of being happy is about twice as high *for both genders* in the most gender-equal countries, compared to the least gender equal.” (p. 523). While most research on gender equality focuses on benefits for women, such as increased professional opportunities, men thus appear to benefit equally from high levels of societal gender equality. Men and women in the most gender-equal societies do not only have a higher well-being; they also have half the chance of being depressed, and about 40 percent less risk of a violent death, compared to men and women living in the least gender-equal societies (Holter 2014). In the United States, similar effects have been found at the state-level (Holter 2014).

Reasons for *why* adult men and women fare so well by high levels of societal gender equality may be sought in a fundamental difference in the social climate in gender-equal vs. gender-unequal societies. Inglehart and colleagues (2002) specified that a society’s prevailing style of social interaction between members of that society changes as societal gender equality increases. In more gender-equal societies, where women are more likely to have an authority role, authority patterns tend to shift from the traditional hierarchical style toward a more collegial style that parallels the differences between stereotypically “male” and “female” styles of social interaction or leadership (Inglehart et al. 2002). That is, while men are more likely to emphasize competition and domination, women tend to have a more cooperative and supportive leadership style. These interaction styles are not limited to the professional context; they are also reflected in other, more private, aspects of social life. Thus, the literature suggests that social climates are more supportive in more gender-equal countries. These supportive social climates may, in turn, positively impact the well-being of adult women ánd men.

Empirical research appears to confirm that countries with relatively high levels of gender equality indeed tend to have more supportive social climates. That is, countries with high levels of gender equality tend to have a cultural preference for (what are traditionally considered) “female” values, such as modesty, cooperation, and support (Cheung and Chan 2007; Hofstede 1998; Schwartz and Rubel-Lifschitz 2009; Ye et al. 2015). In contrast, countries with relatively low levels of gender equality tend to attach more importance to typically “male” values such as achievement, heroism, and assertiveness (Hofstede 1998, 2010). Cultures with a preference for “female” values, or feminine cultures (Hofstede 1998, 2010), typically score higher on well-being, compared to countries with a preference for “male” values, or masculine cultures (Ye et al. 2015). All in all, societal gender equality may affect the well-being of the inhabitants of a country by fostering a more socially supportive climate for all.

### Gender Equality and Life Satisfaction among Adolescents

While the link between gender equality and life satisfaction for adults has been confirmed, much less is known about the impact of gender equality on adolescent life satisfaction. A study by Torsheim and colleagues (2006) touched upon this issue and found that adolescent boys and girls in relatively gender-equal countries reported lower levels of health complaints, compared to boys and girls in relatively gender-unequal countries. The mechanisms explaining this relationship, however, have remained poorly understood and have been hardly addressed in the literature. Based on the assumption that societal gender equality fosters a more socially supportive environment, the hypothesis can be formulated that societal gender equality affects adolescent health and well-being through increased levels of social support in the three most important social contexts of adolescents’ life: the family, the peers, and the school context.

Within the family, high levels of societal gender equality may translate into parents sharing the care of their children and domestic work (Ray et al. 2008). This may affect children’s life satisfaction in three different ways. First, fathers become more involved in child rearing, both in practical and emotional ways (Haas and Hwang 2008; Miller and Sassler 2012; O’Brien 2009). Fathers who are more involved in child rearing have better relationships with their children (Cabrera and Tamis-LeMonda 2013; Sarkadi et al. 2008; Wilson and Prior 2011), and this translates into higher well-being of these children (Wilson and Prior 2011). Second, the parental sharing of child care tasks and domestic work has been linked to higher maternal well-being (Galtry and Callister 2005), presumably because mothers feel supported in combining the child care tasks with a professional career. It has also been related to higher paternal well-being, potentially because caring for children has a buffering effect on psychosocial symptoms for men working long hours (Krantz et al. 2005). Higher parental well-being, in turn, translates into higher adolescent well-being (Powdthavee and Vignoles 2008). Finally, the parental sharing of child care tasks and domestic work has been related to improved relationship satisfaction and higher couple stability among mothers and fathers (Kalmijn 1999; Schober 2013). A positive family climate and stable family structure, in turn, are among the strongest predictors of adolescent life satisfaction (Bjarnason et al. 2012; Diener and Diener McGavran 2008; Levin and Currie 2010; Proctor et al. 2009; Viner et al. 2012).

Gender equality may exert an influence outside of the family as well. The social climate in schools and among peers is likely to be more supportive and less competitive in countries with higher levels of gender equality, compared to countries with lower levels of gender equality. While research on the association between societal gender equality and school climate is scarce, research has pointed out that educational systems in feminine cultures tend to value social adaptation (rather than academic performance), friendliness in teachers (rather than their brilliance), and mutual solidarity (rather than competition) (Hofstede et al. 2010). Negative social behaviors, such as bullying, are less common (Inchley et al. 2016) and appear to be less accepted (Ciby and Raya 2015; Hofstede et al. 2010) in countries with feminine cultures, compared to masculine cultures. Importantly, the characteristics of the school climate on which countries with feminine cultures score typically higher (i.e., high teacher and classmate support), have been consistently associated with better mental health outcomes in adolescents (García-Moya et al. 2015; Ottova et al. 2012). In contrast, the focus on achievement and competition in masculine cultures may increase school-related stress in some adolescents. This, in turn, may translate into a lower well-being (Ottova et al. 2012).

In sum, adolescents in more gender-equal countries may benefit from a more supportive social climate in the family, peer, and school context, compared to adolescents in less gender-equal countries. With social support being one of the strongest predictors of life satisfaction of youth (e.g., Diener and Diener McGavran 2008; Proctor et al. 2009; Viner et al. 2012), it can be hypothesized that social support in the family, peer, and school context explains the positive association between societal gender equality and adolescent life satisfaction.

### Does Gender Equality Impact Boys ánd Girls?

One of the most well-established findings in well-being research is that girls tend to report lower well-being than boys (Cavallo et al. 2006; Inchley et al. 2016; Torsheim et al. 2006). Yet, studies addressing links between gender equality and life satisfaction among adults (Holter 2014) suggest that men as well as women benefit from high levels of societal gender equality. Moreover, an abundance of literature shows that males as well as females benefit from a more socially supportive climate (e.g., Chu et al. 2010; Diener and Diener McGavran 2008; Viner et al. 2012). Therefore, it can be expected that adolescent boys and girls benefit equally from societal gender equality.

## Current Study

Despite a large body of literature documenting consistent cross-national differences in adolescent life satisfaction in Europe and North America, these cross-national differences remain poorly understood. In contrast to previous studies that predominantly focused on the explanatory role of economic factors, this study takes a more social approach. Based on the assumption that high levels of societal gender equality foster a more socially supportive climate for all, the current study examines whether adolescents living in more gender-equal countries have a higher life satisfaction, compared to their peers in less gender-equal countries. Using a large, cross-national dataset including 34 European and North American countries, we addressed the following research questions: (1) Is societal gender equality associated with adolescent life satisfaction?; (2) Is this association explained by social support in the family, peer and school context?; and (3) Is this association equally strong for boys and girls? We predict that gender equality can (partly) explain cross-national differences in adolescent life satisfaction in Europe and North America, over and above economic factors. Specifically, we hypothesize that this association can be explained by social support from parents, peers, and classmates. Finally, in line with empirical findings on adult samples in Europe and North America (e.g., Holter 2014) and based on evidence showing that males as well as females benefit from a more socially supportive climate (e.g., Chu et al. 2010; Diener and Diener McGavran 2008; Viner et al. 2012), we expect that the life satisfaction of adolescent boys ánd girls is higher in countries with higher levels of societal gender equality.

## Methods

### Study Population and Procedures

We used survey data collected in the 2009/10 cycle of the Health Behaviour in School-aged Children (HBSC) study (Currie et al. 2012). Anonymous surveys were conducted in the classrooms of 11-, 13- and 15-year olds according to a common research protocol. A clustered sampling design was used, where the initial sampling unit was the school. Samples were representative geographically, with variations in sampling criteria permitted to fit country-level circumstances. Some countries oversampled subpopulations (e.g., by geography, ethnicity), and therefore survey weights were applied.

In total, 34 countries were included in the analysis (*N* = 175,470; for an overview of the countries please see Table 1). The total sample consisted of 52% girls, *M*_age_ = 13.6 years old (*SD* = 1.64). Each participating country obtained approval to conduct the survey from the ethics review board or equivalent regulatory body associated with their respective institutions/countries. Participation was voluntary and informed consent was sought from school administrators, parents and children according to local human subject requirements. School response rates varied by country but were >70% in most countries. At the student-participant level, response rates ranged from 44 to 92%, but they were >70% in almost all countries. For more information on study procedures, see Roberts and colleagues (2009).

**Table 1:** Descriptive statistics

		Individual-level characteristics							National-level characteristics		
Country	N	Adolescent life satisfaction	% Living with biological parents	% Very easy to talk to mother	% Very easy to talk to father	% Very easy to talk to best friend	Liking school (scale 0–3)	Classmate support (scale 0–4)	Gender empowerment measure	GNI per capita	Gini-index
Armenia	4425	8.52	93.99	45.11	24.19	49.67	2.47	3.24	0.412	5.450	0.31
Austria	1339	7.58	74.10	41.52	24.92	48.18	2.10	2.93	0.744	39.390	0.29
Belgium	6730	7.59	71.22	34.74	18.50	40.55	1.87	2.99	0.874	37.800	0.33
Canada	12754	7.34	67.44	39.63	22.22	51.77	1.92	2.69	0.830	37.280	0.33
Croatia	5904	7.53	87.95	56.61	32.30	49.92	1.43	2.95	0.618	18.730	0.34
Czech Rep.	3831	7.51	68.71	42.59	22.84	46.52	1.87	2.75	0.664	23.640	0.26
Denmark	3475	7.53	66.18	45.47	25.60	48.11	2.07	3.11	0.896	40.290	0.25
Estonia	3890	7.67	66.51	41.37	14.80	44.67	1.54	2.88	0.665	19.510	0.36
Finland	6175	7.77	73.67	43.79	25.87	47.01	1.79	2.77	0.902	37.180	0.27
France	5377	7.53	72.47	33.19	16.24	51.06	1.97	2.81	0.779	34.440	0.33
Germany	4385	7.40	76.75	37.88	22.14	43.24	2.15	3.12	0.852	38.140	0.28
Greece	4420	7.85	86.68	46.70	24.80	58.09	1.72	2.62	0.677	27.380	0.34
Hungary	4381	7.41	72.94	41.05	26.11	52.65	2.01	2.90	0.590	19.270	0.31
Iceland	9844	7.97	71.19	56.99	38.81	49.79	2.31	3.04	0.859	28.720	–
Ireland	3690	7.60	76.89	44.14	26.91	51.66	1.81	2.96	0.722	32.520	0.34
Italy	4396	7.53	82.42	38.50	19.54	52.03	1.66	3.04	0.741	31.130	0.36
Latvia	3452	7.33	64.53	39.80	19.64	45.65	2.01	2.72	0.648	16.350	0.36
Lithuania	4504	7.56	69.47	41.04	19.69	38.56	2.13	2.70	0.628	17.870	0.38
Luxembourg	3328	7.65	76.06	41.93	25.24	47.83	1.74	3.05	–	63.950	0.31
Netherlands	4044	7.99	80.03	55.25	34.40	47.73	2.18	3.08	0.882	42.610	0.31
Norway	3760	7.78	73.16	43.85	26.53	46.29	2.23	3.16	0.906	57.100	0.26
Poland	3795	7.22	81.38	48.48	29.16	50.75	1.72	2.76	0.631	19.010	0.34
Portugal	3388	7.49	78.44	38.79	20.93	53.04	1.94	3.15	0.753	24.710	0.38
Romania	4232	7.57	75.95	54.18	32.58	47.80	2.20	2.87	0.512	14.060	0.31
Slovakia	4184	7.45	77.48	37.42	21.80	51.22	1.66	2.79	0.663	23.120	0.26
Slovenia	4948	7.67	83.80	52.49	35.61	59.39	1.81	3.08	0.641	27.140	0.31
Spain	4303	7.96	81.55	45.45	26.25	65.86	1.76	3.14	0.835	31.640	0.35
Sweden	5832	7.72	76.96	47.32	32.62	50.43	2.00	3.16	0.909	39.660	0.25
Switzerland	5924	7.70	78.77	39.28	20.91	50.24	1.83	3.07	0.822	48.960	0.34
Turkey	4409	6.65	83.24	46.33	23.95	58.54	2.23	2.92	0.379	15.180	0.43
Ukraine	4830	7.22	74.40	46.91	20.81	49.83	2.12	2.88	0.461	6.560	0.28
Macedonia	3024	8.13	86.30	59.91	42.77	54.09	2.38	3.28	0.641	10.830	0.44
UK	12967	7.50	65.19	46.27	26.47	51.15	1.98	2.80	0.790	36.590	0.36
USA	5588	7.52	59.45	40.46	24.02	51.70	1.99	2.71	0.767	47.120	0.41
**Mean**	**171528**	**7.58**	**74.28**	**44.55**	**25.73**	**50.18**	**1.95**	**2.92**	**0.713**	**29.501**	**0.33**

Russia is excluded from the analyses because of missing information on living with both parents

Missing information at the national level was substituted by the mean

– no information available

## Measures

### Dependent Variable (Individual Level)

#### Life satisfaction

Life satisfaction was measured by Cantril’s (1965) ladder, asking students to rate how they presently feel about their life on a ladder ranging from 0, representing the worst possible life, to 10, representing the best possible life. Minor wording change was conducted on the original item to facilitate its use in 11 year olds in the HBSC study. This reworded scale has a good reliability and convergent validity among adolescents in the ages between 11 and 15 years old (Levin and Currie 2014). Although measures such as the (adapted) Cantril ladder may be susceptible to contextual factors (e.g., language effects, cultural measurement bias), these factors cannot explain the observed cross-cultural differences in life satisfaction (Tov and Diener 2009). The Cantril ladder can thus be used as a valid and reliable measure of life satisfaction across nations (Veenhoven 2012).

### Background Characteristics (Individual Level)

#### Gender

The participants indicated their gender by responding to the item “Are you a boy or a girl?” Responses were 0 (boy) and 1 (girl).

#### Age

Age was derived from an item recording a participant’s month and year of birth and relating that to the date of survey administration.

#### Family affluence

The HBSC Family Affluence Scale (FAS; Currie et al. 2008b) was used as a proxy for socioeconomic status. The HBSC 2009/10 survey used a four-item assessment of common material assets or activities of an adolescent’s family (e.g., “Does your family own a car, van or truck?” with response options: no, one, two or more / “How many computers does your family own?” with response options: none, one, two, more than two). Responses were scored and summed. In this analysis, adolescents’ relative socioeconomic position in society was calculated by comparing the individual’s summary score from the FAS to all other scores in the respective country. The ridit-based relative affluence score was used to identify groups of young people in the lowest 20% (low affluence), middle 60% (medium affluence) and highest 20% (high affluence) in each country. By equalizing the distribution of low, medium and high relative family affluence, we effectively disregard country differences in absolute poverty and material standards of living (Inchley et al. 2016). In the current sample, the average score across countries on the FAS variable before transformation into a relative measure was 9.85 (*SD* = 1.98). Norway had the highest average score on FAS (11.24) while the lowest average FAS score was found in Turkey (7.14).

#### Family structure

Family structure was assessed by one question, asking respondents to indicate who resides in the home they live all or most of the time. Answers were recoded as follows: 1 = living with both biological parents; 2 = living with biological mother only; 3 = living with biological father only; and 4 = living with neither biological parent. Research indicates that adolescents living with both biological parents have higher life satisfaction compared to adolescents living in other family structures (Bjarnason et al. 2012).

### Social Support (Individual Level)

#### Ease of communication with parents and best friend

Participants responded to three single-item measures asking about how easy it is to talk with their mother/father/best friend about things that really bother them (answering categories ranging from 1 = very easy to 4 = very difficult and 5 = I don’t have or see a mother/father/best friend). The items were recoded into dummy variables, with very easy communication being the reference category. Easy and open communication with parents and friends has been identified as a protective factor against poor health outcomes (Moreno et al. 2009) and is associated with higher life satisfaction among adolescents across countries (Levin and Currie 2010; Levin et al. 2011b).

#### Liking school

Participants were asked to answer the question “How do you feel about school at present?” (Answering categories ranging from 1 = I like it a lot, to 4 = I don’t like it at all). This variable has been found to be a powerful correlate of health behaviors and health perceptions across countries (Samdal et al. 1998). For the purpose of this study, it was reversely coded with a higher score reflecting a more positive feeling towards school.

#### Classmate support

Classmate support was assessed by three items indicating the extent to which classmates were experienced as supportive, e.g., “Most of the students in my class are kind and helpful” (Response categories going from 1 = completely agree to 5 = completely disagree). Cronbach’s alpha was 0.71. This is a cross-nationally valid and reliable scale for adolescents (Torsheim et al. 2010). The items were recoded so that a higher score indicated greater classmate support.

### National Characteristics (Macro-Level)

#### Gender equality

In this study, the Gender Empowerment Measure (GEM) was included as a measure of gender equality (UNDP 2009; http://hdr.undp.org). The GEM assesses women’s share in political participation, decision making power, economic participation and command over resources. Thus, it examines the extent to which women are able to actively participate in economic and political life and take part in decision-making. It is calculated by the United Nations Development Programme, using a linear combination of women’s and men’s percentage shares of parliamentary seats, women’s and men’s percentage shares of positions as legislators, senior officials and managers, and women’s and men’s percentage shares of professional and technical positions, and share of economic resources as measured by women’s and men’s estimated earned income.

One criticism of the GEM is that it delivers higher gender inequality outcomes for poor countries (regardless of their actual gender equality situation) because it relies heavily on women’s absolute income (Hawken and Munck 2013). However, this study focuses on European countries and does not include extremely poor countries. Moreover, we deliberately chose the GEM as it measures the proportion of women in management and decision-making positions. According to our hypothesis, this aspect of gender equality is closely linked to a preference for female social interaction styles and a more supportive social climate in societies, and consequently to a higher adolescent life satisfaction. Other indices that focus more on gender (in)equality in life expectancy, education, or labor force participation, like the Gender Development Index (UNDP), Gender Equity Index (Social Watch) and Global Gender Gap Index (World Economic Forum), were therefore considered less relevant.

#### National wealth and income inequality

Estimates of Gross National Income (GNI) per capita and income inequality (Gini index) were available from the World Bank (2010). GNI per capita represents the sum of gross value added by all resident producers in the economy plus product taxes and minus any subsidies not included in the value of the products, divided by the mid-year population, and standardized to US dollars. The Gini index represents the distribution of income among everyone in a society, and ranges theoretically from 0 (*where all persons have equal income*) to 1 (*where one person has all the income and the rest have none*). National wealth and income inequality are traditionally examined together in social models of health (e.g., Elgar et al. 2017).

### Analysis

We estimated linear multilevel regression models to test our expectations. The mixed model function in SPSS 22.0 was used for the model estimation. Multilevel regression models take into account the hierarchical clustering of individuals within countries.

First, we modelled direct associations between life satisfaction and the individual and national characteristics included in this study. In Model A, we computed an empty model without any predictors to estimate the total variance in life satisfaction between individuals and between countries. In Model B, we added information on socio-demographics (age and gender) and family structure, to see whether variation presented in Model A can be explained by differences in sociodemographic factors and family composition between individuals and countries. In Model C, we included the GEM. In Model D, we added GNI per capita and the Gini index to see whether the association between the GEM and life satisfaction could be attributed to wealth and income inequality. In Model E, we included the ease of communication, liking school, and classmate support (at the individual level) in order to examine if these variables explained the association between GEM and life satisfaction. If associations between social support and adolescent life satisfaction are significant and if the association between the GEM and adolescent life satisfaction decreases or disappears, we can conclude that social support (partly) explains the association between the GEM and life satisfaction. Finally, in Model F we tested the hypothesized interaction effect (GEM × gender) to assess whether the association between gender equality and adolescent life satisfaction varied by adolescent gender.

## Results

Table 1 presents the descriptive statistics of our sample. Adolescents, on average, gave their life quite a high grade, *M = *7.58 (*SD* = 1.89). Across countries, most scores were between 7.00 and 8.00, except in three countries (i.e., Armenia and Macedonia score above 8; Turkey scores below 7). Across countries, 74% of the adolescents lived with both biological parents, 20% only or mainly lived with their mother, 3% only or mainly lived with their father, and 3% lived with neither parent. Large differences were observed between countries. To illustrate, 94% of the children in Armenia lived with both biological parents, vs. 59% of the children in the United States. The percentages of adolescents reporting very easy communication with mothers (45%) and best friends (50%) were higher than the percentage of adolescents reporting very easy communication with fathers (26%). Finally, adolescents reported to like school relatively much (*M* = 1.95, *SD* = 0.88 on a scale from 0 to 3) and they rated their classmates as relatively nice (*M* = 2.92, *SD* = 0.77 on a scale from 0 to 4).

In terms of national characteristics, Scandinavian countries scored highest on gender equality (Finland, Norway and Sweden had a score > .90), whereas Turkey scored lowest (.38). The GNI per capita of countries ranged from 5.450 (Armenia) to 63.950 (Luxembourg). Gini indices ranged from .26 (Norway; low income inequality) to .44 (Macedonia, high income inequality).

Table 2 presents the results of the linear multilevel regression analysis of life satisfaction on individual and national characteristics. Model A (the empty model) shows that significant national variance in life satisfaction exists, but the variance is relatively small (0.091/(0.091 + 3.510) = 0.025, meaning that 2.5% of total variance in life satisfaction is located at the national level.

**Table 2:** Results of linear multilevel regression analyses of life satisfaction on individual and national characteristics

	Model A: empty model		Model B: +individual background characteristics		Model C: +gender equality		Model D: +GNI per capita and Gini index		Model E: +social support	
	*B*	*S.E*.	*B*	*S.E*.	*B*	*S.E*.	*B*	*S.E*.	*B*	*S.E*.
Constant	7.597***	0.052	9.964***	0.062	9.550***	0.250	9.486***	0.490	6.790***	0.454
*Background characteristics*										
Gender (female = 1)			−0.207***	0.009	−0.207***	0.009	−0.207***	0.009	−0.175***	0.008
Age			−0.187***	0.003	−0.187***	0.003	−0.187***	0.003	−0.087**	0.003
Relative family affluence										
Low			Ref.	Ref.	Ref.	Ref.	Ref.	Ref.	Ref.	Ref.
Middle			0.411***	0.012	0.411***	0.012	0.411***	0.012	0.301***	0.011
High			0.703***	0.014	0.703***	0.014	0.703***	0.014	0.516***	0.013
Family structure										
Lives with both parents (ref.)			Ref.	Ref.	Ref.	Ref.	Ref.	Ref.	Ref.	Ref.
Only/mainly lives with mother			−0.409***	0.011	−0.409***	0.011	−0.409***	0.011	−0.248***	0.011
Only/mainly lives with father			−0.599***	0.027	−0.599***	0.027	−0.599***	0.027	−0.433***	0.025
Lives with neither parent			−0.514***	0.027	−0.514***	0.027	−0.514***	0.027	−0.354***	0.025
*National characteristics*										
Gender Empowerment Measure (GEM)					0.576*	0.338	0.981*	0.523	0.736	0.484
Gross National Income (GNI) per capita							−0.006	0.005	−0.001	0.005
Gini index							−0.002	0.010	0.001	0.010
*Social support at the individual level*										
Talking with father										
Very easy									Ref.	Ref.
Easy									−0.170***	0.012
Difficult									−0.450***	0.014
Very difficult									−0.794***	0.018
Don’t have or see father									−0.449***	0.014
Talking with mother										
Very easy									Ref.	Ref.
Easy									−0.267***	0.010
Difficult									−0.656***	0.014
Very difficult									−1.016***	0.022
Don’t have or see mother									−0.435***	0.029
Talking with best friend										
Very easy									Ref.	Ref.
Easy									−0.034***	0.009
Difficult									−0.150***	0.016
Very difficult									−0.131***	0.024
Don’t have or see best friend									−0.232***	0.025
Liking school									0.360***	0.005
Classmate support									0.403***	0.006
Variance components										
Individual variance	3.500***	0.012	3.296***	0.011	3.296***	0.011	3.296***	0.011	2.793***	0.010
National variance	0.090***	0.022	0.082***	0.020	0.075***	0.018	0.072***	0.018	0.062***	0.015

*N individual* = 171,528; *N national* = 34

*p < 0.1; **p < 0.05; ***p < 0.01

In Model B, individual-level socio-demographics (age and gender) and family structure were added to the model. In line with previous studies, life satisfaction is higher for boys than for girls, for younger adolescents compared to older adolescents, and for adolescents who live with both biological parents, compared to adolescents who only live with one parent or with neither parent. There is a decrease in national variance in life satisfaction after including these characteristics (from 0.091 to 0.083) which indicates that the cross-national variation in life satisfaction can be partly explained by differences in gender, age and family structure across countries.

In Model C, the societal GEM measure was added. The GEM is positively and significantly associated with life satisfaction, indicating that adolescent life satisfaction is higher in countries with more gender equality. National variance decreases after inclusion of gender equality (from 0.083 to 0.076), meaning that the variation in gender equality explains part of the differences in life satisfaction across countries.

In Model D, national wealth and income equality were added. Results show that these characteristics cannot explain the association between equality and adolescent life satisfaction. This association remains significant and even increases in strength after controlling for national wealth and income inequality, indicating a suppression effect of the economic indicators. Moreover, GNI and the Gini index are not independently associated with life satisfaction if the GEM is taken into account.

Next, we tested whether supportive social relationships in the family, peer, and school context explained the association between societal gender equality and adolescent life satisfaction (Model E). The estimates indicate that adolescents who find it (very) difficult to talk with their father, mother or best friend, or who do not have or see a father, mother or best friend, report significantly lower life satisfaction than adolescents for whom it is very easy to talk with their father, mother or best friend. Also, adolescent life satisfaction is higher for adolescents who like school and who perceive their classmates as supportive. The association between gender equality and life satisfaction was no longer significant after taking into account these variables. This suggests that gender equality affects life satisfaction through supportive social relationships in the family, peer, and school context. Both individual- and national-level variance in life satisfaction were reduced substantially (from 3.355 to 2.828 and from 0.073 to 0.063, respectively) by taking social support into account, meaning that social support at the individual level is a key factor in explaining cross-national differences in adolescent life satisfaction.

Finally, in Model F, the cross-level interaction effect (GEM × gender) was added to the model. The interaction was not significant (*B* = −0.196, *SE* = 0.141), indicating that the association between societal gender equality and life satisfaction is equally strong for boys and girls.

We performed three sensitivity analyses (not reported in the tables) to check the robustness of our results. First, as the GEM is highly correlated with both GNI per capita (Pearson’s *r* = .855, *p* < .01) and the Gini index (Pearson’s *r* = .−0.319, *p* < .1), we conducted additional analyses in which the GNI per capita and Gini index were added in separate models to Model B. By doing this, we could see if the GNI per capita and the Gini index were associated with adolescent life satisfaction if the GEM was not taken into account. The results of these analyses were also non-significant (*B = *.004, *SE* = .003 for GNI per capita; *B* = −.005, *SE* = .010 for the Gini index). This confirms that GNI per capita and the Gini index are not associated with adolescent life satisfaction, and suggests that the results presented in Table 2 are not due to multicollinearity issues among the macro-level variables.

Second, we re-estimated our models excluding Armenia and Turkey from the sample. As can be derived from Table 1, these countries could act as outliers due to their relatively high (Armenia) and low (Turkey) average life satisfaction scores. After excluding Armenia and Turkey, the association between the GEM and adolescent life satisfaction became even stronger (Model D; *B* = .916, *SE* = .394, *p* = .027). In our main analyses we have decided not to exclude Armenia and Turkey in order to present conservative estimates of the relationship between gender equality and life satisfaction.

Third, we estimated models in which the five social support variables were used as dependent variables to examine which dimension of social support is most likely to explain the relationship between the GEM and adolescent life satisfaction. We found that the GEM is most strongly related to classmate support, with a higher GEM being associated with higher levels of classmate support. This suggests that, out of our five social support indicators, the association between gender equality and adolescent life satisfaction is best explained by a positive class environment and supportive interactions between classmates.

## Discussion

Although differences across countries in norms and values have been speculated to play a role in cross-national differences in adolescent well-being (Inchley et al. 2016), little quantitative research has explored this proposition. In the current study, we analysed data from 34 countries in Europe and North America to assess the relationship between societal gender equality and adolescent life satisfaction. The results of our study demonstrate that adolescents living in relatively gender-equal countries report a higher life satisfaction, compared to their peers in less gender-equal countries. This association holds for boys as well as girls, and it was explained by the perception of higher levels of social support within the family, peer and school context among adolescents living in relatively gender-equal countries.

While the link between gender equality and adult well-being has been confirmed in the literature, the link between *adolescent* well-being and gender equality has been hardly addressed. An exception to this is a study by Torsheim and colleagues (2006), who examined associations between gender equality and adolescent health complaints. They found that adolescent boys and girls in relatively gender-equal countries reported lower levels of health complaints, compared to boys and girls in relatively gender-unequal countries. As life satisfaction is more than the absence of health complaints, our study adds to the literature that gender equality not only protects young people against negative health outcomes, but also increases the likelihood of high life satisfaction. Moreover, this study tests mechanisms through which gender equality may impact adolescent life satisfaction. Finally, by including national economic factors (GNI per capita and the Gini index) in our model, we demonstrate that gender equality is associated with adolescent well-being *over and above* economic factors.

Our finding that not national wealth, but societal gender equality is a more important predictor of adolescent life satisfaction in Europe and North America, is in line with some recent empirical studies on adult populations. While worldwide studies indicate that people are happier in wealthier countries, the effect of wealth on life satisfaction appears to fade when only relatively wealthy countries are considered (Diener and Biswas-Diener 2002; Diener et al. 2010; Helliwell Layard and Sachs 2017). According to the theory of Evolutionary Modernization (Inglehart and Norris 2003; Inglehart et al. 2002), this occurs because people’s values, life strategies, and conditions for happiness change as they move from low to high levels of economic and physical security. In line with Maslow’s (1954) need hierarchy, people in relatively wealthy countries attach greater importance to issues such as self-actualization, human rights, morality and equality—as opposed to (greater) economic growth. Consequently, these factors become more important determinants of life satisfaction than economic ones.

Gender equality can be considered an important—if not “*the* most central component” (Inglehart et al. 2002; p. 15)—of value change in postindustrial societies. For adolescents specifically, gender equality may contribute to life satisfaction as it generates “a culture of trust and tolerance” (p. 15), in which feminine values such as cooperation, modesty and social support have a more central place, as compared to traditional masculine values such as competition, assertiveness, and achievement. Children who are raised in cultures with predominantly feminine values, learn to be tolerant towards each other, to care for each other, and to respect self-expression and individual freedom. In turn, they may know they can expect the same from others. This is reflected in their perceptions of how easily they can talk to their parents or best friend about sensitive issues, the extent to which they like school, and the extent to which they rate their classmates as supportive. In the light of all the social, physical, and emotional transitions adolescents go through (e.g., striving for more autonomy from parents; intensifying peer contacts; exploring identity), having a socially supportive network within the family, peer, and school context is a very powerful asset.

Our finding that gender equality affects life satisfaction equally for boys and girls is also in line with findings on adult samples (Holter 2014; Kawachi et al. 1999). Although the belief that gender equality is a women’s—or in this case a girls’—issue is still widely held, gender equality involves men and boys as well (Kimmel 2000). Boys’ lower life satisfaction in relatively gender-unequal countries is not due to a direct reduction of their opportunities, but appears to be related to relatively low levels of perceived social support in their environment. The finding that not only girls, but also boys benefit from gender equality parallels the “spirit level” argument posed by Wilkinson and Pickett (2010) regarding income inequality in societies. This argument states that a society with low income inequality works better for all (not just for the poor), because smaller inequalities within societies increase trust, improve health, and lower crime and violence. The benefits of societal gender equality may work in a similar fashion.

This study should be interpreted with knowledge of its limitations. First, our data remain inherently correlational in nature; thus, we encourage readers to refrain from making causal conclusions about the associations between gender equality and adolescent life satisfaction. Given that we measure gender equality as reported by the UNDP (and not adolescents themselves), we do however avoid some potential problems of endogeneity (i.e., life satisfaction influencing adolescents’ perceptions of gender equality). Moreover, previous research at the individual level has shown that, although attitudes on gender equality and well-being seem to have some mutual effect on each other, the main causal chain seems to run from gender equality to well-being (Holter et al. 2009). A second limitation is a potential selectivity bias in our sample due to our use of complete case analysis. Given that non-respondents are likely from higher risk groups, we possibly have slightly inflated estimates of adolescent life satisfaction. Reducing variability in the sample may result in an underestimation of the association between predictors and life satisfaction. Third, a critical question is whether measures of adolescent life satisfaction are valid and reliable across cultures. Self-reports may be vulnerable to contextual factors (e.g., question wording and order effects, translation effects, different response styles; Diener et al. 2013). Yet, even though these factors may play a role, they are unlikely to explain the substantial cultural differences that have been observed (Tov and Diener 2009). It might also be an over-simplification to view countries as contextual units homogeneous within themselves with regard to the dominance of certain social standards. However, given the way currently-available data sets and indicators of gender inequality are constructed, they appear to be a reasonable unit of analysis for empirical work. Finally, a fourth limitation entails that we were not able to control for some potentially important predictors of adolescent life satisfaction at the individual level, such as personality characteristics and intrapersonal competences (Proctor et al. 2009), due to the limited availability of measures in the HBSC dataset. If future datasets become available that include such measures, then replications of our analyses including these factors would be warranted. Future research may examine the confounding or moderating role of such individual-level characteristics, as well as focus more extensively on mechanisms through which societal gender equality may affect adolescent well-being, for example by looking at national social policies supporting gender equality within the family and by measuring actual involvement of mothers and fathers in child care tasks.

This study also has a number of strengths. First, the use of large, nationally representative samples and the inclusion of 34 countries increase the generalizability of our findings. Second, by applying multilevel analyses, we take into account the hierarchical clustering of individuals within countries. This approach is conservative, especially compared to existing research on the link between national characteristics and life satisfaction, which is largely based on correlational studies with aggregated data (Diener et al. 2009). Finally, the simultaneous inclusion of both economic (GNI per capita and Gini index) and social (gender equality) national factors in this study is innovative; previous studies have rarely analysed social and economic indicators of health and well-being in a single design (Ahnguist et al. 2012; Ottova et al. 2012).

## Conclusion

Cross-national differences in adolescent life satisfaction in Europe and North America are consistent, but remain poorly understood. In contrast with previous studies that predominantly focused on the explanatory role of economic factors, this study takes a more social approach. It demonstrates that adolescents have a higher life satisfaction when living in countries with high levels of gender equality, compared to countries with low levels of gender equality—irrespective of individual and national economic factors. While some people still believe that gender equality is a women’s or girls’ issue, this study clearly shows that not only girls, but also boys benefit from higher levels of societal gender equality. The association between societal gender equality and adolescent life satisfaction was explained by the perception of social support within the family, peer and school context. These findings underscore the importance of building a society that promotes equality and recognizes the value of social support for its individual members.
